# Activated ROCK/Akt/eNOS and ET-1/ERK pathways in 5-fluorouracil-induced cardiotoxicity: modulation by simvastatin

**DOI:** 10.1038/s41598-020-71531-8

**Published:** 2020-09-07

**Authors:** Radwa Nasser Muhammad, Nada Sallam, Hanan Salah El-Abhar

**Affiliations:** 1 Department of Pharmacology and Toxicology, Faculty of Pharmacy, Cairo University, Cairo, 11562 Egypt; 2 Department of Pharmacology & Toxicology, Faculty of Pharmaceutical Sciences and Pharmaceutical Industries, Future University in Egypt, Cairo, 11835 Egypt

**Keywords:** Molecular biology, Biomarkers, Cardiology, Oncology

## Abstract

5-Fluorouracil (5-FU) is used in the treatment of different solid tumors; however, its use is associated with rare, but serious cardiotoxicity. Nevertheless, the involvement of ROCK/NF-κB, Akt/eNOS and ET-1/ERK1/2 trajectories in the cardiotoxic effect and in the potential cardioprotective upshot of simvastatin has been elusive. Male Wistar rats were allocated into 5-FU (50 mg/kg/week; i.p, 6 weeks), simvastatin (15 mg/kg/day; p.o, 8 weeks) treated groups and simvastatin + 5-FU, besides the normal control group. 5-FU-induced cardiotoxicity boosted the serum level of N-terminal pro-brain (B-type) natriuretic peptide (NT-proBNP), aortic contents of endothelin (ET)-1 and thromboxane (TX) A2, as well as cardiac contents of NADPH oxidases (Nox), cyclooxygenase (COX)-2, malondialdehyde (MDA), phosphorylated Akt (*p*-Akt), phosphorylated extracellular signal-regulated kinase (*p*-ERK)1/2 and the protein expressions of rho-kinase (ROCK) and caspase-3. On the other hand, it suppressed cardiac reduced glutathione (GSH) and phosphorylated endothelial nitric oxide synthase (*p*-eNOS). Contrariwise, co-administration with simvastatin overcame these disturbed events and modulated the ROCK/NF-κB, Akt/eNOS and ET-1/ERK1/2 signaling pathways. This study highlights other mechanisms than coronary artery spasm in the 5-FU cardiotoxicity and reveals that NT-proBNP is a potential early marker in this case. Moreover, the cross-talk between ROCK/ NF-κB, ROS/COX-2/TXA2, Akt/eNOS and ET-1/ERK1/2 pathways contributes via different means to upsetting the vasoconstriction/vasodilatation equilibrium as well as endothelial cell function and finally leads to cardiomyocyte stress and death—the modulation of these trajectories offers simvastatin its potential cardio-protection against 5-FU.

## Introduction

Cardio-oncology has recently gained wide recognition as it explores the relationship between the two clinical disciplines; oncology and cardiology. The main objective of cardio-oncology is to respond to the needs of patients who receive cancer therapy and to promote research for the development of effective cancer treatment methods, while minimizing cardiovascular (CV) events^1^.

One of the widely used antineoplastic drugs in solid tumors is 5-fluorouracil (5-FU), which is a cornerstone therapy in colorectal cancer (CRC)^2,3^. Although the drug is generally well-tolerated, it also possesses a number of common toxicities, including gastrointestinal upsets, myelosuppression and neurotoxicity^4,5^, as well as cardiotoxicity^6,7^. This type of toxicity is less frequently reported and, unfortunately, not often appreciated although it can be potentially serious and even lethal^8^. Roth et al*.*^9^ first reported the 5-FU-associated cardiotoxicity, which has an incidence that can range up to 35% depending on the dose, the dosing regimen, cardiac comorbidity, and susceptibility of patients^10^. Hence, the reported frequency substantially differs from one study to another. Although 5-FU is the second most common cause of chemotherapy-induced cardiotoxicity after anthracyclins, it is quite different in terms of its cardiac side effects^8^.

The clinical presentation of cardiotoxicity caused by 5-FU is very diverse and wide, but it appears mainly as angina-like transient myocardial ischemia in most clinical reports^11–13^. Nonetheless, the reported cardiac side effects range from silent electrocardiographic (ECG) changes up to myocardial infarction, besides congestive heart failure, cardiomyopathy, pericarditis, hyper- and hypo-tension, ventricular and supraventricular tachycardia, QT-prolongation, sino-atrial and atrio-ventricular nodal dysfunction, cardiogenic shock, and sudden death^2,14^.

Nevertheless, it is not clinically easy to handle this type of cardiotoxicity because the associated pathophysiological and molecular mechanisms are still poorly investigated. Moreover, the number of animal or laboratory studies conducted until now is not sufficient. Based on those few studies, a number of mechanisms were suggested, including coronary artery spasm^15^, endothelial damage^16,17^, thrombogenic effects^18^, and direct myocardial toxicity-induced necrosis^2,19^. Furthermore, the accumulation of toxic metabolites, the occurrence of myocardial autoimmune-mediated injury, the disruption of the tri-carboxylic acid cycle within cardiomyocytes and the diminished ability of red blood cells to transfer oxygen^7^ were also reported. However, acute myocardial ischemia caused by coronary artery spasm was the most commonly reported and is the most accepted theory up till now^7,8^.

In the context of cardiac ischemia and cardiomyocyte injury, it might appear that the gold standard biomarkers, namely, cardiac troponins (cTns), are a reliable index^20^. Yet, the results were inconsistent with 5-FU, and the serum level of these cardiac structural proteins might not be trustworthy to reflect the severity of the case^21^.

Increasing attention has been directed towards other emerging cardiac injury-related markers, the natriuretic peptides, including B-type (or brain) natriuretic peptide (BNP) and the inactive N-terminal fragment (NT-proBNP). More studies evaluated the utility of BNP and NT-proBNP in the diagnosis and prognosis of patients who are suspected of having an acute coronary event^22^. In fact, as the size and severity of the ischemic insult increase, plasma BNP emerges at high rates with acute coronary ischemia. Even though the sensitivity and specificity are likely to be insufficient, both peptides are promising prognostic markers in acute coronary syndromes^23^. Moreover, the escalation of these peptides correlates with the severity of aortic stenosis and cardiac ischemia/reperfusion injury^24,25^. Interestingly, it was reported that BNP elevation correlates with suppressing the gene expression of endothelin (ET)-1 and prevents the stimulation of several hypertrophic kinases, such as protein kinase C (PKC)^26^.

Besides, accumulating evidence suggests that the Rho-kinase (ROCK) system, identified twenty years ago, may play an important role in the development of CV disease (CVD) through augmenting oxidative stress, inflammation, endothelial cell (EC) injury, as well as contraction and proliferation of vascular smooth muscle cell (VSMC)^27^. In a feedforward scenario, the expression of ROCKs and their activity in human coronary VSMCs can be up-regulated by inflammatory stimuli via PKC and nuclear factor-kappa B (NF-κB)-dependent pathways^28^. Moreover, ROCKs are important negative upstream regulators of endothelial nitric oxide (NO) synthase (eNOS) in ECs^29^. In a mutual effect, the suppression of endothelial NO potentiates ROCK activity in coronary arteries^30^ and the inhibition of ROCK pathway leads to rapid phosphorylation and activation of eNOS via the phosphatidylinositol-3 kinase (PI3K) /protein kinase B (PKB/Akt) pathway^31^.

Apart from their lipid lowering effect, different members of the 3-hydroxy-3-methyl-glutaryl-CoA (HMG-CoA) reductase inhibitors, or commonly statins family, demonstrated their cardioprotective upshot through their pleiotropic properties in both pre-clinical and clinical studies, a merit that is partly related to the inhibition of ROCKs^29,32,33^.

Accordingly, we aim at evaluating the possible involvement of some trajectories in the 5-FU-induced CV injury and the potential aptitude of simvastatin (Sim) to protect the heart against this chemotherapeutic drug.

## Methods

### Animals

Adult male albino Wistar rats weighing 230–250 g were obtained from the animal facility of the National Research Centre, (Cairo, Egypt). Rats were housed under controlled environmental conditions at temperature of 25 ± 2 °C, humidity conditions (60 ± 10%), constant light cycle (12 h light/dark), and allowed free access to standard chow and water^34^. The investigation complies with the Guide for Care and Use of Laboratory Animals published by the US National Institute of Health (NIH Publication No. 85–23, revised 2011) and was approved by the Ethics Committee for Animal Experimentation at Faculty of Pharmacy, Cairo University (Permit Number: PT 1637).

### Drugs

5-FU and Sim were obtained from S.X. Haipu Pharmaceutical Co. (Shanghai, China) and Merck Co. (NJ, USA), respectively.

### Pharmacological treatments

After a two-week acclimation period, animals were randomly allocated into four groups (n = 15–20 per group). In the first control group, rats received normal physiological saline (2 ml/kg i.p) once weekly for six successive weeks to serve as the normal control group, whereas animals in the second control group were daily gavaged Sim (15 mg/kg/day)^35^ for eight successive weeks. Rats in the third group received 5-FU (50 mg/kg; i.p) once weekly for six successive weeks to serve as the cardiotoxic group and finally, those in the fourth group received Sim for one week before the first 5-FU injection, then concomitantly for six weeks, and continued alone for another week after the last dose of 5-FU. A fresh suspension of Sim was daily prepared in sterile water^35^. The dose and the dosing schedule of 5-FU were chosen based on a pilot study that depended on dosage conversion factors, toxicological studies of 5-FU, and colon cancer treatment regimens in humans^36–39^. Animals were daily recognized for food and water intake and mortality, and were weekly weighed to monitor weight loss.

### ECG measurements

One hour after each 5-FU injection, five to six rats from each group were randomly chosen and anaesthetized with thiopental (50 mg/kg, i.p.; Sigma-Aldrich Co., MO, USA). Rats were kept warm with a heating lamp to avoid the risk of hypothermia. Subcutaneous peripheral limb electrodes were inserted for ECG recording (HPM 7100, Fukuda Denshi, Tokyo, Japan) to determine heart rate (HR), QTc and RR intervals, QRS duration, and to test for ST-segment elevation^34^.

### Preparation of samples

At the end of the experimental period, animals were weighed then anaesthetized using ketamine/xylazine (60/7.5 mg/kg, i.p) and blood was collected from the femoral vein using non-heparinized capillary tubes for separation of sera. Afterwards, animals were euthanized by cervical dislocation under anesthesia to minimize suffering, and the whole heart and thoracic aortic tissues were rapidly excised, washed, dried and weighed. The aorta was used as an alternative to the coronary arteries due to limited access to rat coronaries and difficulty of their separation, in addition to the very small tissue size. Different studies used the aorta and other arteries to study the toxic effect of 5-FU^15,17,18^.

From each group, heart tissues and the isolated thoracic aortae of six rats were used for histopathological examination, whereas the rest (n = 6–10/group) were used for cardiac/aortic biochemical assessments. In the latter groups, the heart/rat was incised longitudinally and the first part was homogenized in ice-cold saline to prepare 10% homogenate. The other part was used for the preparation of 67% homogenate in RIPA buffer for western blotting analysis.

### Biochemical analysis

#### Serum levels of cTnI and NT-proBNP

According to the manufacturer, the serum levels of cTnI and NT-proBNP were measured using the corresponding ELISA kits. The kit for cTnI was procured from Kamiya biomedical company, (WA, USA; Cat# KT-478) and that for NT-proBNP was obtained from MSD, (MD, USA; Cat# K153JKD).

#### Cardiac contents of p-NF-κB p65, phosphorylated extracellular signal-regulated kinase (p-ERK1/2), p-Akt and p-eNOS

The corresponding ELISA kits were used to determine the cardiac contents of *p*-NF-κB p65 (Ser536) (Abcam, Cambridge, UK; Cat# ab176647), *p*-ERK1/2 (Thr202/Tyr204) (Invitrogen, CA, USA; Cat # EMS2ERKP), *p*-Akt (Ser473) (Elabscience, Wuhan, China; Cat # E-EL-R1135) and *p*-eNOS (Ser1177) (Cell Signaling Technology, MA, USA; Cat# 7980C).

#### Aortic contents of ET-1 and thromboxane (TX) A2

The MyBioSource (California, USA) ELISA kits were used to quantitatively estimate the aortic contents of ET-1 (Cat # MBS704215) and TXA2 (MBS267231). All ELISA kits were used following the manufacturers’ instructions. The aortic content of ET-1 was measured rather than the plasma, since the latter is affected by some irrelevant factors in human^40^.

#### Cardiac contents of NADPH oxidase (Nox), cyclooxygenase (COX)-2, reduced glutathione (GSH) and malondialdehyde (MDA)

For the determination of cardiac contents of Nox, COX-2, GSH and MDA, the corresponding commercial kits were used according to the manufacturers’ procedures. The source and catalogue number appear in the parenthesis as follows: Nox (LifeSpan Biosciences, WA, USA; Cat # LS-F5720), COX-2 (Cusabio, Wuhan, China; Cat # CSB-E13399r), GSH (Biodiagnostic, Cairo, Egypt; Cat# GR 25 11) and MDA (Biodiagnostic, Cairo, Egypt; Cat # MD 25 29).

#### Cardiac caspase-3 activity and DNA fragmentation analysis

The activity of cardiac caspase-3 was assessed with the corresponding colorimetric assay kit (R&D Systems Inc, MN, USA; Cat. # K106-100). Absorbance was read at 405 nm and results were expressed as nmol pNA/h/mg protein. In addition, DNA fragmentation analysis was carried out to reflect the apoptotic process on genomic DNA integrity, where 30 mg of ground heart tissue were used for DNA extraction and purification using Gentra Puregene® DNA purification kit (Qiagen, Hilden, Germany; Cat. # 158667). The purity and yield of the extracted DNA were assessed using NanoDrop® spectrophotometer (ThermoFisher Scientific, MA, USA). Finally, 10 µg of DNA per well was run in an electrophoresis chamber using 1.5% agarose gel and ethidium bromide. A molecular weight marker DNA (Sigma-Aldrich, MO, USA) was loaded to facilitate comparison with the samples. DNA ladders were visualized and photographed under UV light.

### Western blot analysis of ROCK and caspase-3

Total cardiac protein content was extracted, using the corresponding protein extraction kit (Bio-Rad, CA, USA), then quantified for protein levels using Bicinchoninic acid protein assay kit (ThermoFisher Scientific, MA, USA). Following total protein extraction, equal amounts (20 μg) of protein were separated by electrophoresis according to their molecular weights on SDS-PAGE, then electro-transferred to nitrocellulose membranes (Bio-Rad, CA, USA). The protein expression was assessed as previously described by Ahmed et al*.*^41^. Concisely, the membranes were incubated overnight with primary antibodies against ROCK (1:1,000, R&D systems, MN, USA; Cat. # AF4790-SP), caspase-3 (1:1,000, MyBioSource, CA, USA; Cat. # MBS9382732) or β-actin (1:1,000, ThermoFisher Scientific, MA, USA; Cat. # PA1-183). Afterwards, the membranes were washed and incubated with horseradish peroxidase (HRP)-conjugated secondary antibody (1:2,000; ThermoFisher Scientific, MA, USA). The blots were finally developed using the enhanced chemiluminescence reagent (Pierce™ western ECL substrate; ThermoFisher Scientific, MA, USA) and signals were detected using a charge-coupled device camera-based imager (ChemiDoc MP imager; Bio-Rad, CA, USA). Proteins were quantified by the provided image analysis software and results were expressed as arbitrary units after normalization to β-actin expression.

### Histopathological examination

For histological examination, the whole heart and part of the thoracic aorta were separated (n = 6/group), rinsed with ice-cold saline and immediately fixed in 10% formal saline. Specimens were processed for paraffin embedding, and 5 µm sections were prepared^41^. The sections were stained with hematoxylin and eosin (H&E) (Sigma–Aldrich Chemical Co., MO, USA) and examined blindly under light microscope (magnification × 400). Images were captured and processed using Adobe Photoshop (version 8.0)^41^. Cardiac and aortic injury scores were analyzed using the Leica Qwin 500 Image Analyzer (Leica Microsystems, Wetzlar, Germany). Random areas were examined for each group (three sections per animal), where both cardiac and aortic sections were scored from 0 to 4 to describe the severity of each injury hallmark. Score (0) indicated no histopathological lesions, score (1) described a limited focal distribution of lesions, scores (2) and (3) described moderate severity with multiple scattered histopathological lesions, and score (4) labelled the presence of severe lesions over the entire examined sections^42^. The assessed cardiac and aortic injury hallmarks included vacuolation of the sarcoplasm of cardiomyocytes, congestion of myocardial blood vessels, intermuscular edema, intramyocardial inflammatory cells infiltration, focal necrosis of cardiomyocytes and finally, vacuolation of cells of tunica media of the aorta.

### Statistical analysis

Data are expressed as means ± standard error of mean (SEM). Statistical analysis, graphical representations, and regression analysis were performed using GraphPad Prism software, version 6 (GraphPad Software Inc., USA). Results were analyzed using one-way analysis of variance test (ANOVA) followed by Tukey’s multiple comparisons test. The histopathological injury scores were analyzed using the non-parametric Kruskal–Wallis test followed by Dunn's multiple comparison test, and data are expressed as the median and range (min–max). For all statistical tests, the level of significance was fixed at P < 0.05.

## Results

### Effect of Sim on final body weight and mortality in 5-FU treated animals

In this study, it was noticed that animals treated with 5-FU showed severe nasal, rectal, and ocular heamorrhage, ascites, as well as a decrease in food and water consumption, which resulted in a significant decrease in the final body weight (213 ± 11.9 vs 310 ± 14.4 g). The drug also resulted in a 60% mortality. On the other hand, co-treatment with Sim significantly improved food and water consumption, evidenced by the reduced body wasting, and the percentage of mortality was limited to 28%. In addition, 5-FU-associated hemorrhage and ascites were almost absent in Sim co-treated group (Table 1).

**Table 1:** Effect of Sim on 5-FU-induced changes in final body weight and mortality.

	Final body weight (g)	% of mortality
Control	310 ± 14.4	0
Sim	315 ± 9.56	0
5-FU	213 ± 11.9*^,#^	60
5-FU + Sim	289 ± 16.1^@^	28

Values are presented as the mean of 15–20 experiments ± SEM. Statistical analysis was carried out using one-way ANOVA followed by Tukey’s post-hoc test; as compared to normal (*), Sim (#), and 5-FU (@)-treated groups (p < 0.05).

*5-FU* 5-fluorouracil, *Sim* simvastatin.

### Effect of Sim on ECG changes in 5-FU treated animals

**Figure 1:**
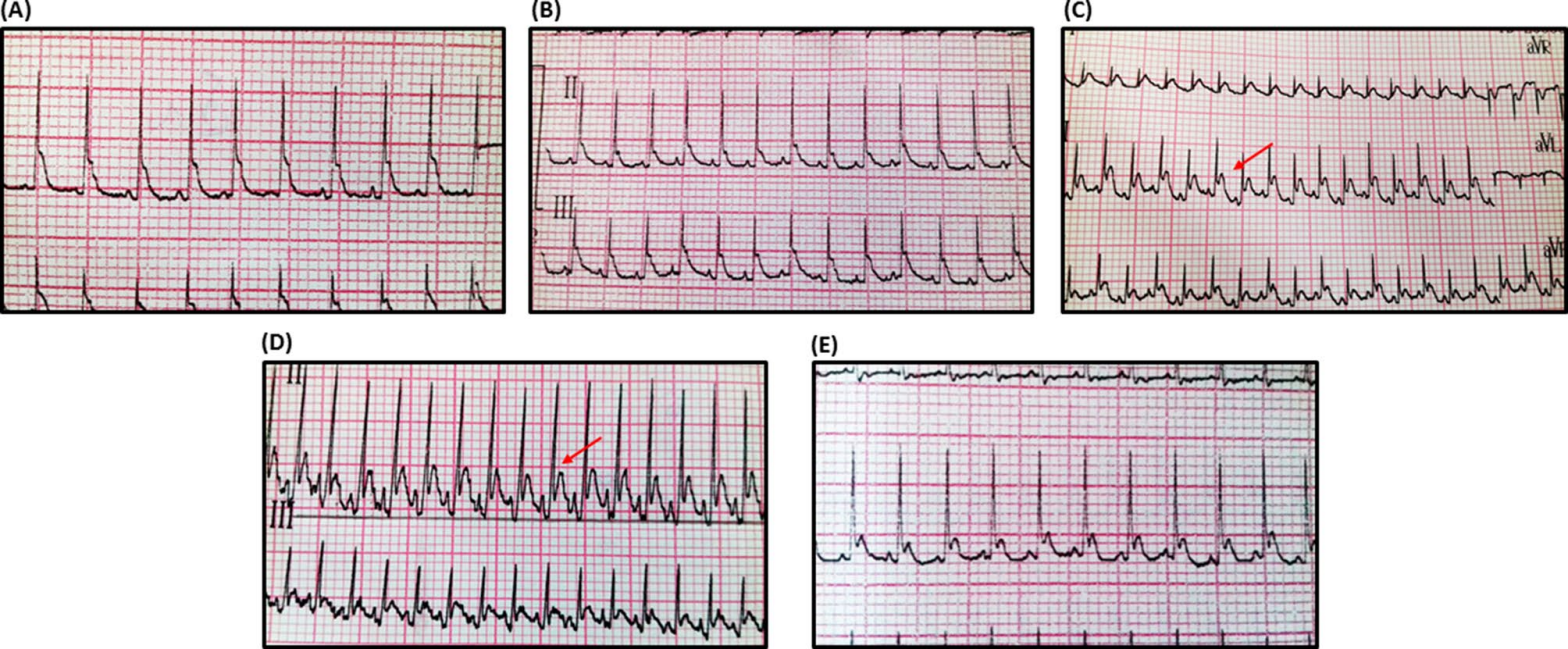
ECG recording from (**A**) Normal control, (**B**) Sim-treated, (**C**, **D**) 5-FU-treated and (**E**) 5-FU + Sim treated groups. ECG shows normal pattern, except in panels (**C**, **D**), which clearly show elevation in the ST segment (red arrow). *5-FU* 5-fluorouracil, *Sim* simvastatin.

The ECG recording (leads II and III) (Fig. 1) depicts the 5-FU-induced abnormalities on cardiac electrophysiology. It caused a two-fold elevation in the ST segment and prolonged the QTc duration to reach about 1.1 fold, with no remarkable effect on the QRS duration, as compared to the ECG of normal control rats. Additionally, 5-FU treatment prominently peaked the T-wave, caused a 13% percent drop in HR and about 15% prolongation in the RR interval duration, as compared to the control group. Interestingly, Sim was able to normalize most of the 5-FU-induced ECG alterations, with no substantial effect on either HR or RR interval (Table 2).

**Table 2:** Effect of Sim on 5-FU-induced changes in ECG findings.

	ST-elevation (mV)	QTc duration (ms)	QRS duration (ms)	RR interval (ms)	HR (bpm)
Control	0.131 ± 0.0078	399 ± 4.90	56.14 ± 1.55	306.5 ± 6.386	194.2 ± 4.347
Sim	0.134 ± 0.0065	382 ± 10.50	54.00 ± 2.70	334.0 ± 4.121	182.9 ± 2.593
5-FU	0.259 ± 0.0149*^,#^	441 ± 7.09*^,#^	62.14 ± 1.68^#^	360.1 ± 7.412*^,#^	171.3 ± 3.259*^,#^
5-FU + Sim	0.122 ± 0.0087^@^	400 ± 5.21^@^	61.14 ± 2.07	347.1 ± 7.262*	170.9 ± 2.167*^,#^

Values are presented as the mean of 10 experiments ± SEM. Statistical analysis was carried out using one-way ANOVA followed by Tukey’s post-hoc test; as compared to normal (*), Sim (#), and 5-FU (@)-treated groups (p ˂ 0.05).

*5-FU* 5-fluorouracil, *bpm* beat per minute, *HR* heart rate, *Sim* simvastatin.

### Cardiac injury and stress biomarkers

**Figure 2:**
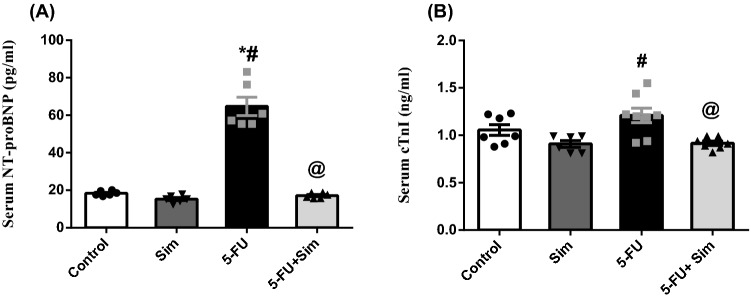
Effect of 5-FU and Sim treatments on serum levels of (**A**) NT-proBNP and (**B**) cTnI. Values are presented as the mean of 6–8 experiments ± SEM. Statistical analysis was done using one-way ANOVA followed by Tukey’s *post-hoc test*; as compared to normal (*), Sim (#), and 5-FU (@)-treated groups (*p* < 0.05). *5-FU* 5-fluorouracil, *cTnI* cardiac troponin I, *NT-proBNP* N-terminal pro-brain natriuretic peptide, *Sim* simvastatin.

As depicted in Fig. 2, administration of 5-FU caused a state of myocardial cell stress evidenced by the significant increase in the serum level of (A) NT-proBNP by more than three folds, as compared to the control group, an effect that was significantly ameliorated in Sim co-treated animals. Contrary to the elevation in NT-proBNP, 5-FU did not show any significant increase in the universal cardiac biomarker (B) cTnI, compared to the normal control group. However, Sim in normal rats caused a subtle insignificant decrease (14%) in the cTnI, compared to the normal group. This effect was interestingly observed in animals receiving 5-FU and co-treated with Sim**.**

### Effect of 5-FU and Sim treatments on aortic contents of ET-1 and TXA2

**Figure 3:**
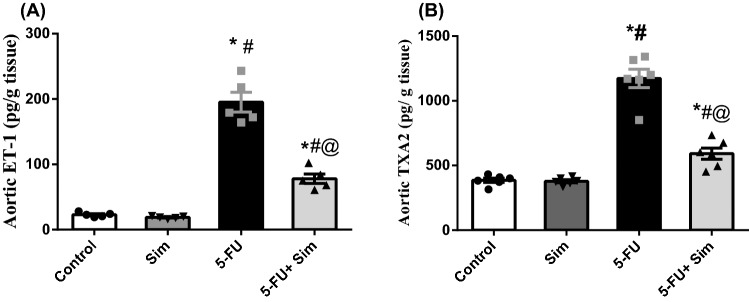
Effect of 5-FU and Sim treatments on aortic tissue contents of (**A**) ET-1 and (**B**) TXA2. Values are presented as the mean of 6 experiments ± SEM. Statistical analysis was done using one-way ANOVA followed by Tukey’s *post-hoc test*; as compared to normal (*), Sim (#), and 5-FU (@)-treated groups (p < 0.05). *5-FU* 5-fluorouracil, *ET-1* endothelin-1, *Sim* simvastatin, *TXA2* thromboxane A2.

In the aortic tissue (Fig. 3), 5 -FU increased the contents of the two potent vasoconstrictor molecules; viz*.*, (A) ET-1 and (B) TXA2 by more than 8 and 3 folds, respectively, compared to the control group. On the other hand, Sim co-administration almost halved these escalations.

### Effect of 5-FU and Sim treatments on cardiac ERK 1/2, ROCK, caspase-3 and genomic DNA integrity

**Figure 4:**
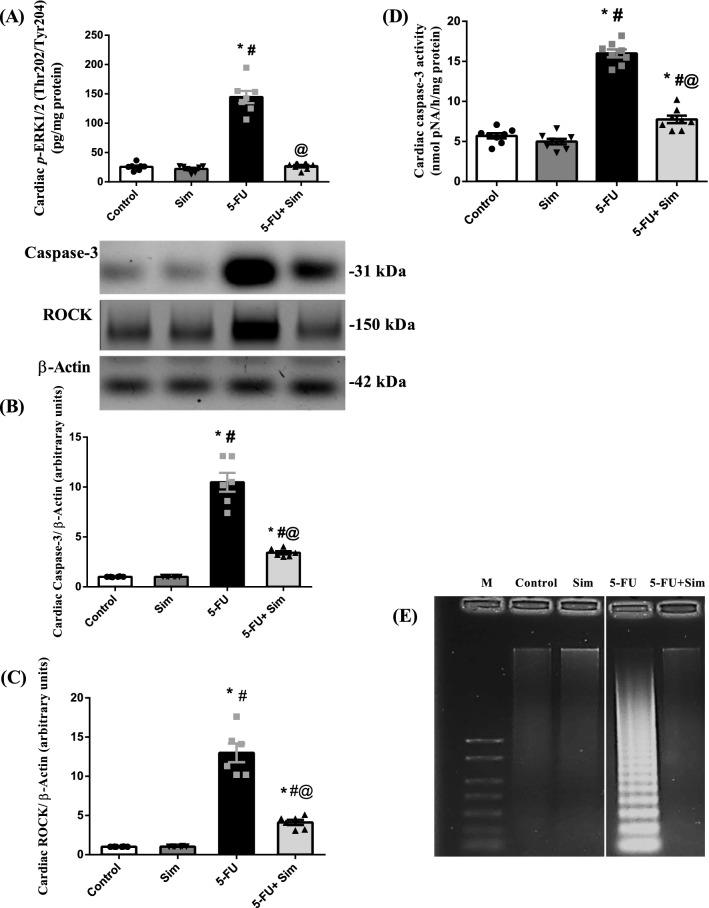
Effect of 5-FU and Sim treatments on cardiac (**A**) content of *p*-ERK, (**B**) expression of ROCK, (**C**) expression and (**D**) activity of caspase-3 and (**E**) DNA fragmentation analysis, in which 5-FU group shows DNA laddering. Values are presented as the mean of 6–8 experiments ± SEM. Statistical analysis was carried out using one-way ANOVA followed by Tukey’s post-*hoc test*; as compared to normal (*), Sim (#), and 5-FU (@)-treated groups (p < 0.05). *5-FU* 5-fluorouracil, *M*, marker DNA (1 kb), *p-ERK1/2 (Thr202/Tyr204)*, phosphorylated extracellular signal-regulated kinase 1/2 at Thr 202 & Tyr 204, *ROCK* rho-kinase, *Sim* simvastatin.

As shown in Fig. 4, 5-FU bolstered the cardiac content of (A) *p*-ERK1/2 by more than 5 folds and sharply (13 folds) induced the protein expression of (B) ROCK, a regulator of calcium ion intake, contractility, and cell death. These effects entailed the apoptotic marker (C) caspase-3 (10 folds), as compared to the normal control animals (see Supplementary Fig. S2). The latter finding was further confirmed by the (D) 81% escalation of its activity. Finally, apoptotic cell death was further confirmed by the (E) marked disruption of intact genomic DNA, characterized by a striking laddering pattern. On the other hand, Sim co-administration thwarted the 5-FU effects and suppressed these upshots markedly.

## Cardiac oxidative and inflammatory status

**Figure 5:**
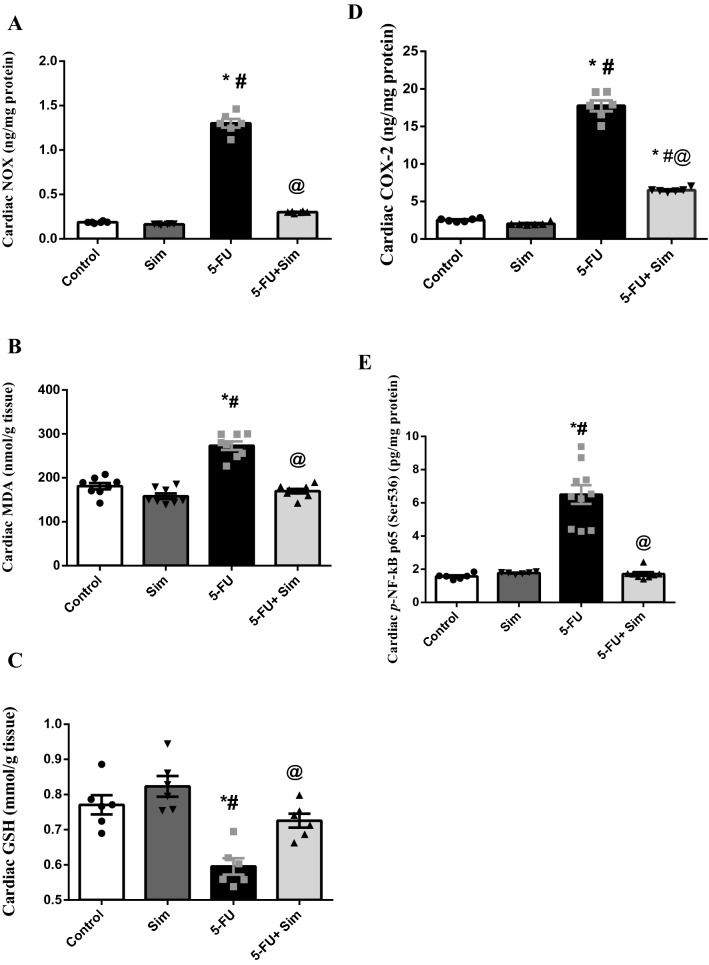
Effect of 5-FU and Sim co-treatment on cardiac tissue contents of (**A**) Nox, (**B**) MDA, (**C**) GSH, (**D**) COX-2 and (**E**) *p*-NF-κB p65. Values are presented as the mean of 6–8 experiments ± SEM. Statistical analysis was carried out using one-way ANOVA followed by Tukey’s post-hoc test; as compared to normal (*), Sim (#), and 5-FU (@)-treated groups (p < 0.05). *5-FU* 5-fluorouracil, *COX-2* cyclooxygenase-2, *GSH* glutathione reduced form, *MDA* malondialdehyde, *p-NF-κB p65 (Ser536)* phosphorylated nuclear factor-kappa B p65 at Ser 536, *Nox* NADPH-oxidase, *Sim* simvastatin.

Administration of 5-FU triggered cardiac oxidative stress (Fig. 5) as manifested by the marked upsurge of (A) total Nox and (B) MDA that were associated with a 23% decrease in (C) GSH. Moreover, 5-FU boosted the inflammatory enzyme (D) COX-2, which enhanced its downstream molecule (E) *p*-NF-κB p65, compared to the control group. However, to signify its antioxidant and anti-inflammatory properties, Sim reduced Nox, lipid peroxidation, COX-2 and *p*-NF-κB p65, but replenished the defence molecule GSH.

### Effect of 5-FU and Sim treatments on cardiac contents of p-Akt and p-eNOS

**Figure 6:**
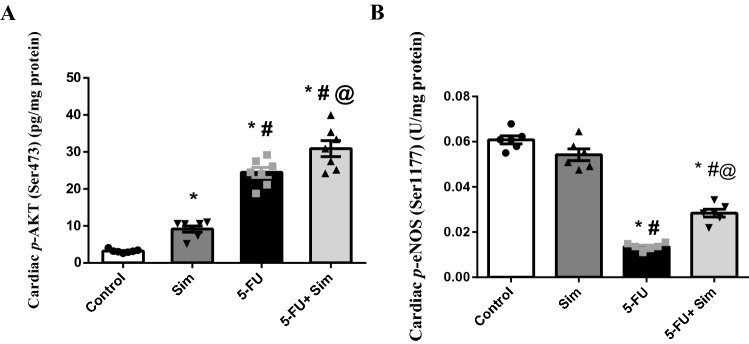
Effect of 5-FU and Sim treatments on cardiac tissue contents of (**A**) *p*-Akt and (**B**) *p*-eNOS. Values are presented as the mean of 6–7 experiments ± SEM. Statistical analysis was carried out using one-way ANOVA followed by Tukey’s *post-hoc test*; as compared to normal (*), Sim (#), and 5-FU (@)-treated groups (p < 0.05). *5-FU* 5-fluorouracil, *p-Akt (Ser473)* phosphorylated protein kinase B at Ser 473, *p-eNOS (Ser1177)* phosphorylated endothelial nitric oxide synthase at Ser 1177, *p-ERK1/2 (Thr202/Tyr204)* phosphorylated extracellular signal-regulated kinase 1/2 at Thr 202 & Tyr 204, *Sim* simvastatin.

As illustrated in Fig. 6, 5-FU triggered the cardiac tissue content of (A) *p*-Akt by more than seven-folds, compared to the normal control group, an effect that was further escalated upon treatment with Sim. It is noteworthy to mention that Sim in the normal animals also elevated this kinase by 3 folds relative to the normal group. However, this picture was the opposite in the Akt down-stream target; where 5-FU sharply declined the cardiac content of (B) *p*-eNOS by about 80%, as compared to the control group, to be reverted by the administration of Sim.

### Histopathological findings in the heart and aorta

**Figure 7:**
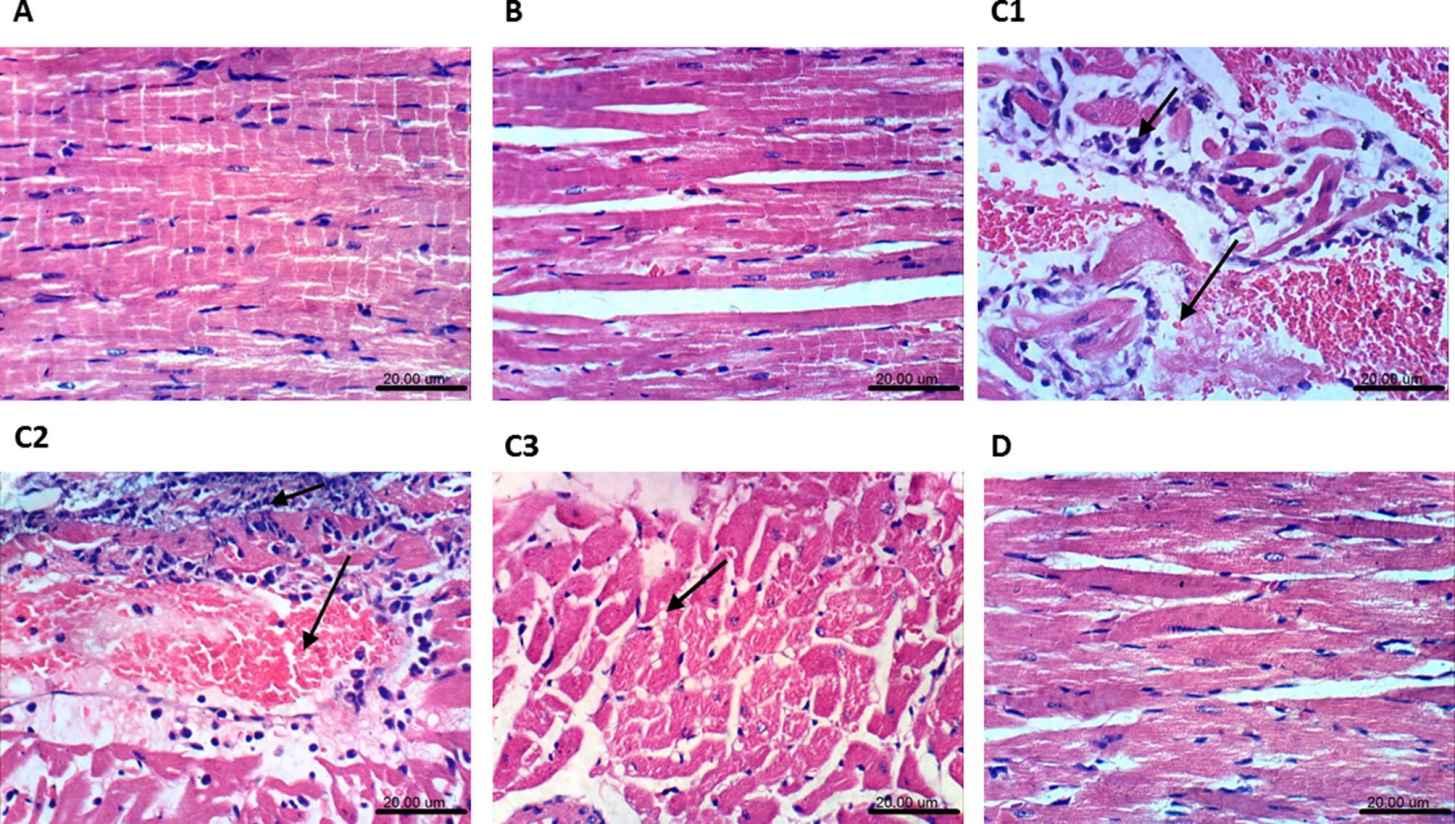
Effect of Sim on 5-FU-induced histological changes in cardiomyocytes. Photomicrographs (**A**–**D**) represent specimens taken from the heart and changes are indicated in the panel by black arrows. Slides of (**A**) normal control group and (**B**) normal animals treated with Sim show unremarkable changes, while that from (**C1**) 5-FU group shows intermuscular inflammatory cells infiltration associated with edema. Additionally, (**C2**, **C3**) slides from 5-FU group show focal necrosis of cardiomyocytes associated with congestion of myocardial blood vessel, as well as vacuolization of the sarcoplasm of cardiomyocytes. However, slide from animals treated with (**D**) 5-FU + Sim shows no histopathological changes. *5-FU* 5-flourouracil, *Sim* simvastatin (H&E × 400; scale bar: 20 µm).

**Figure 8:**
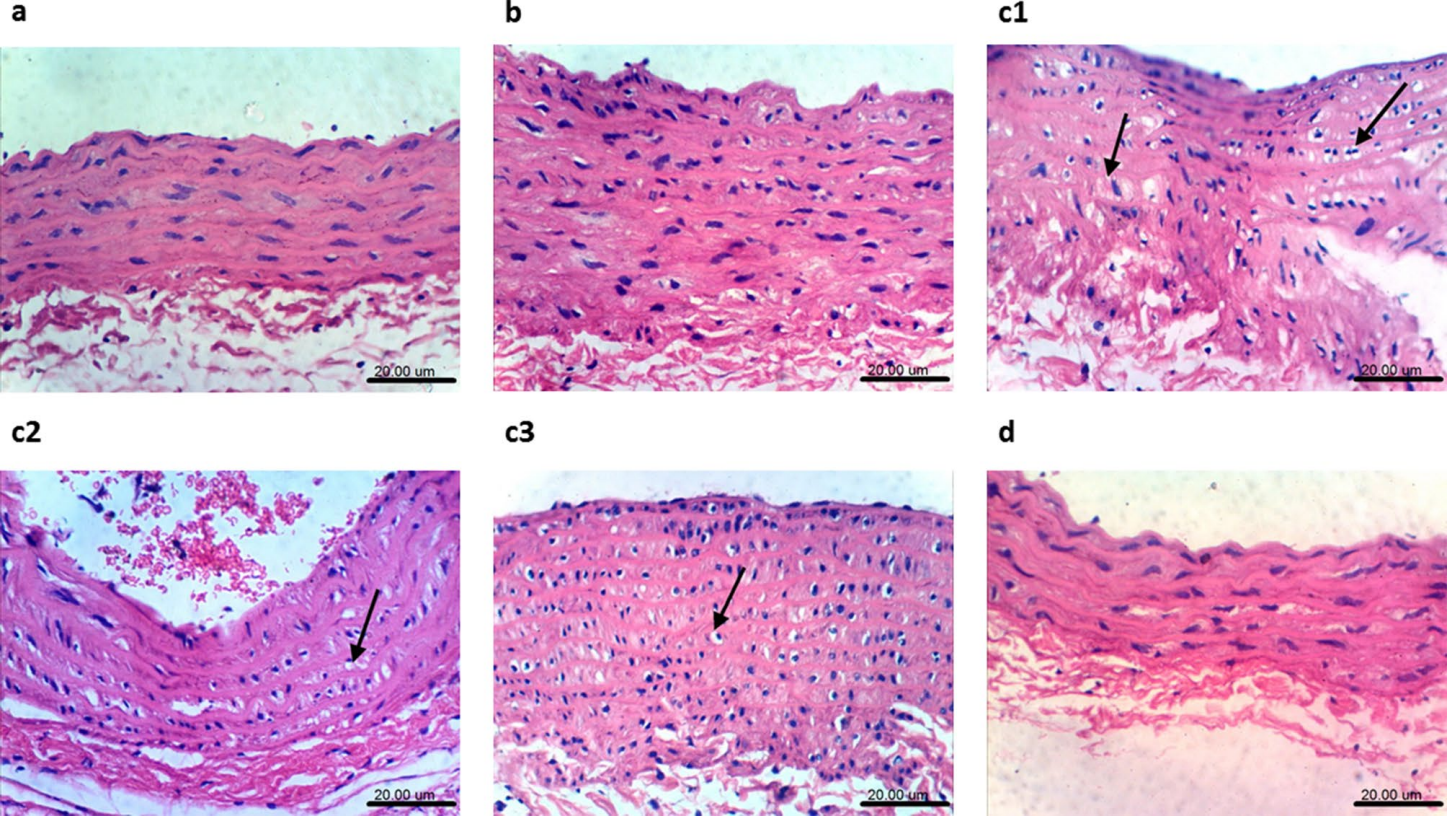
Effect of Sim on 5-FU-induced histological changes in aortic tissue. Photomicrographs from **a-d** represent specimens taken from the aorta. Sections from (**a**) normal control group and that (**b**) treated with Sim, show normal histological layers with no histopathological changes. On the contrary, sections of (**c1**–**c3**) 5-FU treated animals show vacuolation of cells of tunica media, whereas section (**d**) from 5-FU + Sim group shows no histopathological changes. *5-FU* 5-flourouracil; *Sim* simvastatin (H&E × 400; scale bar: 20 µm).

Treatment with 5-FU altered the myocardial structure (Fig. 7), where the (C1–C3) photomicrographs showed vacuolization in the sarcoplasmic tissue, intermuscular edema, congestion of myocardial blood vessels, and focal necrosis of cardiomyocytes associated with inflammatory cells infiltration**.** However, these morphological changes were not detected in the (D) 5-FU + Sim section, guided by the (A) normal structure shown in the normal control group and (B) normal group treated with Sim. Moreover, sections of the aortic tissue (Fig. 8) showed normal structure in both (A) normal control and (B) normal animals treated with Sim**,** whereas (C1–C3) sections of 5-FU-treated group showed vacuolization of cells of tunica media. However, this alteration was not obvious in (D) 5-FU + Sim treated group. In the same context, cardiac and aortic injury scores were markedly improved by Sim treatment (Table 3).

**Table 3:** Effect of Sim on cardiac and aortic injury scores.

	Cardiac injury/lesion score	Aortic histopathology score
Control	0.0 (0–0)	0.0 (0–0)
Sim	0.0 (0–0)	0.0 (0–0)
5-FU	3 (2–4)*^,#^	3.5 (3–4)*^,#^
5-FU + Sim	0.5 (0–1)^@^	0.5 (0–1)^@^

Values (n = 6) are expressed as median (min–max). Statistical analysis was performed using Kruskal–Wallis nonparametric one-way ANOVA followed by Dunn's multiple comparison test; as compared to normal (*), Sim (#), and 5-FU (@)-treated groups (p < 0.05).

*5-FU* 5-fluorouracil, *Sim* simvastatin.

## Discussion

The current study highlights the involvement and crosstalk of several signaling pathways in the 5-FU-induced cardiac injury. These pathways include the ET-1/ERK1/2, ROS/COX-2/TXA2, ROCK/NF-κB, as well as Akt/eNOS which augment the vasoconstriction/vasodilation imbalance and trigger cell demise by apoptosis. The study also substantiates the cardioprotective potential of Sim against 5-FU-induced injury by manipulating these pathways. The histopathological examination and cardiac function tests further support the biochemical findings.

In our study, 5-FU was shown to cause significant weight loss and body wasting, which were associated with high mortality, a result that matches earlier studies showing the anorexic effect of 5-FU in rodents^43^. This effect was partially related to the 5-FU-induced mucositis and the impaired tight junctions^43^, an effect that was possibly ameliorated by Sim^44^, resulting in a better weight gain.

Studying the cardiotoxic effect of 5-FU, our data showed no elevation in the serum level of cTnI, the gold standard of cardiomyocyte injury. However, this unexpected finding concurs with previous studies in which cardiac troponins were not detected in the sera of patients receiving 5-FU, even those in whom a clinical evidence of cardiotoxicity was prominent^14,45^.

Apart from cTnI, the European Cardiology Society has endorsed both NT-proBNP and BNP to be assessed in patients with a history of ACS and in the management of heart failure^46,47^. Our results showed that NT-proBNP, the one with longer half-life and stability^48^, was markedly elevated in 5-FU-treated rats, demonstrating cardiomyocyte injury and stress. This data concurs with earlier studies conducted on patients receiving 5-FU and showing cardiac stretch remodeling^49^. It also supports the studies conducted on patients who received continuous 5-FU infusion^50^ or complained about atypical chest pain two days after the first chemotherapeutic dose^51^. Hence, based on the present work and the clinical data available hitherto, NT-proBNP might be a promising marker for 5-FU cardiotoxicity.

Besides, arterial vasoconstriction induced by 5-FU has been amply reviewed^52,53^, which supports the boosting effect of 5-FU on the potent vasoconstrictor ET-1, and hence matches the findings of Porta et al.^54^ and pins down the theory of 5-FU-induced coronary vasospasm^55^. A positive correlation between ET-1 and NT-proBNP is plausible, since a synchronized elevation between the two markers was reported here and earlier^56^. The increased ET-1 entailed its downstream molecule ERK1/2, which matches previous studies^57,58^ and points to the related hypertrophic responses and endothelial dysfunction^59^; however, the transient spasm of the coronaries cannot solely account for 5-FU cardiotoxicity. Cardiomyocytes injury was confirmed by the histopathological findings reported herein and previously^6,10,60^, with the vacuolization being a prominent feature in both the heart and aorta, indicating degenerative processes^61^ and cardiotoxicity^62^. This was also supported by the altered cardiac electrophysiology, which could reflect an acute myocardial injury.

It is worth mentioning that Sim co-treatment normalized NT-proBNP, ET-1, and *p*-ERK1/2 and prevented most of the functional and histological abnormalities. These findings are consistent with a wide range of studies revealing the cardioprotective effect of statins on cardiac histology following a variety of injurious insults^63,64^. However, scarce data links the cardioprotective effect of Sim to the inhibition of NT-proBNP^65^ or ET-1^66^. In addition, various studies displayed the inhibitory impact of Sim on ERK1/2 activation, phosphorylation and signaling^67–69^, a result that was also supported by the present study.

Moreover, 5-FU is thought to mediate cardiac injury by triggering oxidative stress as reported hitherto^2,5,19,70^; this was confirmed here by the increased Nox enzyme which produces reactive oxygen species (ROS) along with lipid peroxidation, as well as the decreased defense molecule GSH. The exact cause of ROS overproduction was not clear; however, several pathways crosstalk increased ROS production. The 5-FU-associated ET-1 elevation activates Nox^71,72^, and in a feedforward signal, ROS stimulate ET-1 via the MAPK/ERK1/2-dependent pathway to further mediate a negative impact on ECs^73^. Moreover, ROS activate other molecules, such as COX isoforms that also contribute to endothelial dysfunction^73^; all of which are facts that support the current findings. On the other hand, the aptitude of Sim for reducing Nox activity, lipid peroxidation, ET-1/ERK 1/2 signal and COX-2 enzyme, as well as the enhancement of the defense molecule coincides with previous reports^59,74,75^ and highlights its anti-oxidant and anti-inflammatory effects that are partially responsible for its cardioprotective effect against 5-FU.

Besides the redox imbalance, the increased ERK1/2 and COX-2 paralleled with the enhanced TXA2 in the 5-FU-treated group. The current results confirm the interconnected loop between these molecules as reported earlier in a model of hypertension^76^, and in 5-FU-induced permanent damage to hepatic ECs, with TXA2 being the major player^77^.

The vasoconstrictor/vasodilator imbalance detected in the 5-FU-treated group was further substantiated by the low cardiac content of eNOS compared to the augmented vasoconstrictors; viz., ET-1 and TXA2. The modulation of several molecules by 5-FU led ultimately to a decrease in NO, the vasodilator molecule. As reported earlier, NO is reduced following the inhibition of eNOS, and the augmentation of TXA2^76,78^ and ROS^79,80^. Therefore, suppression of the aortic TXA2 in Sim co-treated animals could be attributed to the Sim-mediated suppression of COX-2 and ROS.

Our results also highlighted the role of 5-FU in mediating the activation of the ROCK/NF-κB signaling pathway, which augments vasoconstriction and endothelial dysfunction. Both molecules are linked to the surplus production of ROS^81^, and in a mutual role, Nox-derived ROS stimulate NF-κB signaling^82^. Moreover, the up-regulation of different Nox subunits was responsible for the activation of ROCK that promotes the development of CVD^27,76^. Additionally, the activation of NF-κB and ROCK reduces the stability of eNOS mRNA^83^. Therefore, inhibition of both ROCK and NF-κB in the Sim-treated group further indicates its cardioprotective and vasodilator capacities. Hunter and coworkers^84^ reported that NO mediates an anti-ET-1 effect in cardiomyocytes via the inhibition of the ROCK cascade in the context of cardiac hypertrophy. Moreover, ROCK inhibitors have an outstanding vasodilator activity^27^. Additionally, statins were reported to stabilize eNOS mRNA by the inhibition of Rho geranyl-geranylation, independent from their cholesterol lowering activity^27,73^. These findings, thus, prove the crosstalk between several factors to shape part of the 5-FU-induced CVD scenario and emphasize that their regulation can be a target therapy, as documented here upon administration of Sim.

Apart from their role in ROS production, increasing vascular tone, and endothelial disruption, activated ROCKs end up with cell death^85^. The present study pinned down the apoptotic fate of cardiomyocytes exposed to 5-FU, where it heightened both the expression and activity of caspase-3 to support earlier findings in different models of CVDs^29,85^. The apoptotic process was demonstrated further by the prominent DNA fragmentation. On the other hand, Sim co-treatment completely abolished caspase-3 protein over-activation, partially by inhibiting ROCK to concur with the study of Ahmed et al.^41^.

The PKB or Akt, a downstream effector of the PI3K and an important activator of eNOS^83,86^, is another kinase assessed in this study. Although 5-FU caused a remarkable elevation in this kinase, this was not reflected on its downstream molecule, eNOS. This effect may indicate a potential compensatory mechanism that failed to face the vasoconstrictor mediators. A possible support to this hypothesis is the further elevation of this kinase by Sim co-treatment, compared to 5-FU; an effect that was coupled by an activation in the eNOS and the inhibition of the vasoconstrictor mediators, as stated earlier in this work. Besides the vasodilatory role of Akt, it also has an impact on apoptosis. Our results coincide with previous data stating that statins rapidly activate endothelial Akt at the serine residue 473^87^, thus improving its protein kinase activity to protect against cardiomyocyte apoptosis in ischemia/ reperfusion injury in mice. Moreover, Kureishi et al.^86^ reported that Sim enhanced the phosphorylation/activation of eNOS and inhibited apoptosis in vitro in an Akt-dependent pattern. Again, the Akt/eNOS hub is linked to ROCK signaling, where inhibition of the latter in rat striatal tissues by Sim led to the activation of PI3K/Akt/eNOS signaling pathway with a subsequent increase in the production and bioavailability of endothelial NO and protection against neurodegeneration^41^.

## Conclusion

Based on the current data, it was revealed that coronary vasospasm cannot solely account for all the cardiotoxicity manifestations associated with 5-FU treatment and that the cardiac lesions observed in animals treated with 5-FU demonstrate a direct toxic effect on the myocardium. Despite the obvious contribution of the imbalanced redox system, inflammatory mediators, and apoptotic damage in such injury, the ischemic insult cannot be neglected, since a profound increase in multiple biomarkers and a disruption in the cardiac electrical activity were observed. Moreover, it seems that NT-proBNP, rather than cTnI, is a potential marker for early 5-FU cardiotoxicity and that several trajectories, namely, ROCK/ NF-κB, Akt/eNOS, ET-1/ERK1/2, and ROS/COX-2/TXA2 signaling pathways, intermingle to induce such toxic effect. Hence, Sim through its ability to modulate these cascades can be nominated as a cardioprotective agent against the cardiotoxic effect of 5-FU.

## Supplementary information

Supplementary Information.

Supplementary Legend.

## References

[1] Albini A (2009). Cardiotoxicity of anticancer drugs: the need for cardio-oncology and cardio-oncological prevention. J. Natl. Cancer Inst..

[2] Focaccetti C (2015). Effects of 5-fluorouracil on morphology, cell cycle, proliferation, apoptosis, autophagy and ROS production in endothelial cells and cardiomyocytes. PLoS ONE.

[3] Jensen SA, Hasbak P, Mortensen J, Sørensen JB (2010). Fluorouracil induces myocardial ischemia with increases of plasma brain natriuretic peptide and lactic acid but without dysfunction of left ventricle. J. Clin. Oncol..

[4] Freeman NJ, Costanza ME (1988). 5-Fluorouracil-associated cardiotoxicity. Cancer.

[5] Eskandari MR, Moghaddam F, Shahraki J, Pourahmad J (2015). A comparison of cardiomyocyte cytotoxic mechanisms for 5-fluorouracil and its pro-drug capecitabine. Xenobiotica.

[6] Tsibiribi P (2006). Cardiac lesions induced by 5-fluorouracil in the rabbit. Hum. Exp. Toxicol..

[7] Layoun ME, Wickramasinghe CD, Peralta MV, Yang EH (2016). Fluoropyrimidine-induced cardiotoxicity: manifestations, mechanisms, and management. Curr. Oncol. Rep..

[8] Sorrentino MF, Kim J, Foderaro AE, Truesdell AG (2012). 5-fluorouracil induced cardiotoxicity: review of the literature. Cardiol. J..

[9] Roth A, Kolaric K, Popovic S (1975). Cardiotoxicity of 5-fluorouracil. Cancer Chemother. Rep..

[10] Polk A, Vistisen K, Vaage-Nilsen M, Nielsen DL (2014). A systematic review of the pathophysiology of 5-fluorouracil-induced cardiotoxicity. BMC Pharmacol. Toxicol..

[11] Herrmann J (2020). Adverse cardiac effects of cancer therapies: cardiotoxicity and arrhythmia. Nat. Rev. Cardiol..

[12] Herrmann J (2020). Vascular toxic effects of cancer therapies. Nat. Rev. Cardiol..

[13] Herrmann J (2016). Vascular toxicities of cancer therapies: the old and the new: an evolving avenue. Circulation.

[14] Polk A, Vaage-Nilsen M, Vistisen K, Nielsen DL (2013). Cardiotoxicity in cancer patients treated with 5-fluorouracil or capecitabine: a systematic review of incidence, manifestations and predisposing factors. Cancer Treat. Rev..

[15] Mosseri M, Fingert HJ, Varticovski L, Chokshi S, Isner JM (1993). In vitro evidence that myocardial ischemia resulting from 5-fluorouracil chemotherapy is due to protein kinase C-mediated vasoconstriction of vascular smooth muscle. Cancer Res..

[16] Campia U (2019). Cardio-oncology: vascular and metabolic perspectives: a scientific statement from the American Heart Association. Circulation.

[17] Kinhult S, Albertsson M, Eskilsson J, Cwikiel M (2003). Effects of probucol on endothelial damage by 5-fluorouracil. Acta Oncol..

[18] Kinhult S, Albertsson M, Eskilsson J, Cwikiel M (2001). Antithrombotic treatment in protection against thrombogenic effects of 5-fluorouracil on vascular endothelium: a scanning microscopy evaluation. Scanning.

[19] Durak I (2000). Reduced antioxidant defense capacity in myocardial tissue from guinea pigs treated with 5-fluorouracil. J. Toxicol. Environ. Health A..

[20] Dhingra R, Vasan RS (2017). Biomarkers in cardiovascular disease: statistical assessment and section on key novel heart failure biomarkers. Trends Cardiovasc. Med..

[21] Depetris I (2018). Fluoropyrimidine-induced cardiotoxicity. Crit. Rev. Oncol. Hematol..

[22] Caselli C (2016). Effect of coronary atherosclerosis and myocardial ischemia on plasma levels of high-sensitivity troponin T and NT-proBNP in patients with stable angina. Arterioscler. Thromb. Vasc. Biol..

[23] Deo R, de Lemos JA (2003). B-type natriuretic peptide in ischemic heart disease. Curr. Cardiol. Rep..

[24] Wagner JA (2007). Natriuretic peptides and myocardial oxygen supply-to-demand ratio in patients with aortic stenosis. Eur. J. Clin. Invest..

[25] Abbas SS, Mahmoud HM, Schaalan MF, El-Abhar HS (2018). Involvement of brain natriuretic peptide signalling pathway in the cardioprotective action of sitagliptin. Pharmacol. Rep..

[26] Ritchie RH, Rosenkranz AC, Kaye DM (2009). B-type natriuretic peptide: endogenous regulator of myocardial structure, biomarker and therapeutic target. Curr. Mol. Med..

[27] Shimokawa H, Sunamura S, Satoh K (2016). RhoA/Rho-kinase in the cardiovascular system. Circ. Res..

[28] Hiroki J (2004). Inflammatory stimuli upregulate Rho-kinase in human coronary vascular smooth muscle cells. J. Mol. Cell Cardiol..

[29] Dong M, Yan BP, Yu CM (2009). Current status of rho-associated kinases (ROCKs) in coronary atherosclerosis and vasospasm. Cardiovasc. Hematol. Agents Med. Chem..

[30] Shimokawa H, Takeshita A (2005). Rho-kinase is an important therapeutic target in cardiovascular medicine. Arterioscler. Thromb. Vasc. Biol..

[31] Wolfrum S (2004). Inhibition of Rho-kinase leads to rapid activation of phosphatidylinositol 3-kinase/protein kinase Akt and cardiovascular protection. Arterioscler. Thromb. Vasc. Biol..

[32] Mahalwar R, Khanna D (2013). Pleiotropic antioxidant potential of rosuvastatin in preventing cardiovascular disorders. Eur. J. Pharmacol..

[33] Shimomura M (2016). Acute effects of statin on reduction of angiopoietin-like 2 and glyceraldehyde-derived advanced glycation end-products levels in patients with acute myocardial infarction: a message from SAMIT (Statin for Acute Myocardial Infarction Trial). Heart Vessels..

[34] Mohamed SS, Ahmed LA, Attia WA, Khattab MM (2015). Nicorandil enhances the efficacy of mesenchymal stem cell therapy in isoproterenol-induced heart failure in rats. Biochem. Pharmacol..

[35] Palus S (2013). Simvastatin reduces wasting and improves cardiac function as well as outcome in experimental cancer cachexia. Int. J. Cardiol..

[36] Freireich EJ, Gehan EA, Rall DP, Schmidt LH, Skipper HE (1966). Quantitative comparison of toxicity of anticancer agents in mouse, rat, hamster, dog, monkey, and man. Cancer Chemother. Rep..

[37] Cheeseman SL (2002). A 'modified de Gramont' regimen of fluorouracil, alone and with oxaliplatin, for advanced colorectal cancer. Br. J. Cancer..

[38] Carrato A (2008). Adjuvant treatment of colorectal. Cancer.

[39] Nair AB, Jacob S (2016). A simple practice guide for dose conversion between animals and human. J. Basic Clin. Pharm..

[40] Baretella O, Vanhoutte PM (2016). Endothelium-dependent contractions: prostacyclin and endothelin-1, partners in crime?. Adv. Pharmacol..

[41] Ahmed LA, Darwish HA, Abdelsalam RM, Amin HA (2016). Role of rho kinase inhibition in the protective effect of fasudil and simvastatin against 3-nitropropionic acid-induced striatal neurodegeneration and mitochondrial dysfunction in rats. Mol. Neurobiol..

[42] Dong R, Liu P, Wee L, Butany J, Sole MJ (1992). Verapamil ameliorates the clinical and pathological course of murine myocarditis. J. Clin. Invest..

[43] Song MK, Park MY, Sung MK (2013). 5-Fluorouracil induced changes of intestinal integrity biomarkers in BALB/c mice. J. Cancer Prev..

[44] Fang X, Xu RS (2015). Protective effect of simvastatin on impaired intestine tight junction protein ZO-1 in a mouse model of Parkinson's disease. J. Huazhong Univ. Sci. Technol. Med. Sci..

[45] Salepci T (2010). 5-Fluorouracil induces arterial vasoconstrictions but does not increase angiotensin II levels. Med. Oncol..

[46] Cacko A (2018). Novel biochemical predictors of unfavorable prognosis for stable coronary disease. Medicine.

[47] Ponikowski P (2016). 2016 ESC Guidelines for the diagnosis and treatment of acute and chronic heart failure: The Task Force for the diagnosis and treatment of acute and chronic heart failure of the European Society of Cardiology (ESC) Developed with the special contribution of the Heart Failure Association (HFA) of the ESC. Eur. Heart J..

[48] Weber M, Hamm C (2006). Role of B-type natriuretic peptide (BNP) and NT-proBNP in clinical routine. Heart.

[49] Nadir MA (2012). Improving the primary prevention of cardiovascular events by using biomarkers to identify individuals with silent heart disease. J. Am. Coll. Cardiol..

[50] Dechant C (2012). Acute reversible heart failure caused by coronary vasoconstriction due to continuous 5-fluorouracil combination chemotherapy. Case Rep. Oncol..

[51] Thalambedu N, Khan Y (2019). Fluorouracil (5-FU)-induced cardiomyopathy. Cureus..

[52] Seker M (2018). Role of urotensin-2 in 5-fluorouracil-related arterial vasoconstriction in cancer patients. Oncol. Res. Treat..

[53] Südhoff T (2004). 5-Fluorouracil induces arterial vasocontractions. Ann. Oncol..

[54] Porta C, Moroni M, Ferrari S, Nastasi G (1998). Endothelin-1 and 5-fluorouracil-induced cardiotoxicity. Neoplasma.

[55] Kosmas C (2008). Cardiotoxicity of fluoropyrimidines in different schedules of administration: a prospective study. J. Cancer Res. Clin. Oncol..

[56] Zaucha-Prażmo A, Sadurska E, Drabko K, Kowalczyk JR (2016). Can we find a good biochemical marker of early cardiotoxicity in children treated with haematopoietic stem cell transplantation?. Contemp. Oncol..

[57] Sugden PH, Clerk A (2005). Endothelin signalling in the cardiac myocyte and its pathophysiological relevance. Curr. Vasc. Pharmacol..

[58] Blixt FW (2019). MEK/ERK/1/2 sensitive vascular changes coincide with retinal functional deficit, following transient ophthalmic artery occlusion. Exp. Eye Res..

[59] Nemoto S, Taguchi K, Matsumoto T, Kamata K, Kobayashi T (2012). Pravastatin normalizes ET-1-induced contraction in the aorta of type 2 diabetic OLETF rats by suppressing the KSR1/ERK complex. Am. J. Physiol. Heart Circ. Physiol..

[60] Kumar S, Gupta RK, Samal N (1995). 5-fluorouracil induced cardiotoxicity in albino rats. Mater. Med. Pol..

[61] Dunnick JK, Johnson J, Horton J, Nyska A (2004). Bis (2-chloroethoxy) methane-induced mitochondrial and myofibrillar damage: short-term time-course study. Toxicol. Sci..

[62] Dunnick JK, Lieuallen W, Moyer C, Orzech D, Nyska A (2004). Cardiac damage in rodents after exposure to bis (2-chloroethoxy) methane. Toxicol. Pathol..

[63] Kudo S (2016). SmgGDS as a crucial mediator of the inhibitory effects of statins on cardiac hypertrophy and fibrosis: novel mechanism of the pleiotropic effects of statins. Hypertension.

[64] Li CB (2012). Simvastatin exerts cardioprotective effects and inhibits the activity of Rho-associated protein kinase in rats with metabolic syndrome. Clin. Exp. Pharmacol. Physiol..

[65] Wilkins MR (2010). Simvastatin pulmonary hypertension trial (SiPHT) study group. Simvastatin as a treatment for pulmonary hypertension trial. Am. J. Respir. Crit. Care Med..

[66] Sahebkar A (2015). Lipid and blood pressure meta-analysis collaboration (LBPMC) group. Statin therapy reduces plasma endothelin-1 concentrations: a meta-analysis of 15 randomized controlled trials. Atherosclerosis.

[67] Zhang Z (2013). Simvastatin inhibits the additive activation of ERK1/2 and proliferation of rat vascular smooth muscle cells induced by combined mechanical stress and oxLDL through LOX-1 pathway. Cell Signal..

[68] Sun F (2015). Simvastatin alleviates cardiac fibrosis induced by infarction via up-regulation of TGF-β receptor III expression. Br. J. Pharmacol..

[69] Sironi L (2006). Activation of NF-kB and ERK1/2 after permanent focal ischemia is abolished by simvastatin treatment. Neurobiol. Dis..

[70] Varricchi G (2018). Antineoplastic drug-induced cardiotoxicity: a redox perspective. Front. Physiol..

[71] Sirker A, Zhang M, Murdoch C, Shah AM (2007). Involvement of NADPH oxidases in cardiac remodelling and heart failure. Am. J. Nephrol..

[72] Cao X (2019). Effects of the (Pro)renin receptor on cardiac remodeling and function in a rat alcoholic cardiomyopathy model via the PRR-ERK1/2-NOX4 pathway. Oxid. Med. Cell Longev..

[73] Vanhoutte PM, Shimokawa H, Feletou M, Tang EH (2017). Endothelial dysfunction and vascular disease: a 30th anniversary update. Acta Physiol..

[74] Burma O (2014). Effects of rosuvastatin on ADMA, rhokinase, NADPH oxidase, caveolin-1, hsp 90 and NFkB levels in a rat model of myocardial ischaemia-reperfusion. Cardiovasc. J. Afr..

[75] Cipollone F (2003). Suppression of the functionally coupled cyclooxygenase-2/prostaglandin E synthase as a basis of simvastatin-dependent plaque stabilization in humans. Circulation.

[76] García-Redondo AB (2015). c-Src, ERK1/2 and Rho kinase mediate hydrogen peroxide-induced vascular contraction in hypertension: role of TXA2, NAD(P)H oxidase and mitochondria. J. Hypertens..

[77] Xing X, Xia S (1996). The injury of liver sinusoidal endothelial cells in a rat's isolated liver perfusion (ILP) model for regional chemotherapy. Zhonghua Wai Ke Za Zhi (CJS).

[78] Liu CQ (2009). Thromboxane prostanoid receptor activation impairs endothelial nitric oxide-dependent vasorelaxations: the role of Rho kinase. Biochem. Pharmacol..

[79] Chandra S (2012). Oxidative species increase arginase activity in endothelial cells through the RhoA/Rho kinase pathway. Br. J. Pharmacol..

[80] Félétou M, Vanhoutte PM (2006). Endothelial dysfunction: a multifaceted disorder (The Wiggers Award Lecture). Am. J. Physiol. Heart Circ. Physiol..

[81] Madamanchi NR, Runge MS (2013). Redox signaling in cardiovascular health and disease. Free Radic. Biol. Med..

[82] Montezano AC (2008). Aldosterone and angiotensin II synergistically stimulate migration in vascular smooth muscle cells through c-Src-regulated redox-sensitive RhoA pathways. Arterioscler. Thromb. Vasc. Biol..

[83] Vanhoutte PM, Zhao Y, Xu A, Leung SW (2016). Thirty years of saying NO: sources, fate, actions, and misfortunes of the endothelium-derived vasodilator mediator. Circ. Res..

[84] Hunter JC (2009). Nitric oxide inhibits endothelin-1-induced neonatal cardiomyocyte hypertrophy via a RhoA-ROCK-dependent pathway. J. Mol. Cell Cardiol..

[85] Sebbagh M (2001). Caspase-3-mediated cleavage of ROCK I induces MLC phosphorylation and apoptotic membrane blebbing. Nat. Cell Biol..

[86] Kureishi Y (2000). The HMG-CoA reductase inhibitor simvastatin activates the protein kinase Akt and promotes angiogenesis in normocholesterolemic animals. Nat. Med..

[87] Fujio Y, Nguyen T, Wencker D, Kitsis RN, Walsh K (2000). Akt promotes survival of cardiomyocytes in vitro and protects against ischemia-reperfusion injury in mouse heart. Circulation.

