# Supplementary material for: Activated ROCK/Akt/eNOS and ET-1/ERK pathways in 5-fluorouracil-induced cardiotoxicity: modulation by simvastatin
Source: Sci Rep. 2020 Sep 7;10:14693. doi: 10.1038/s41598-020-71531-8 (PMC7477553; doi:10.1038/s41598-020-71531-8)

Activated ROCK/Akt/eNOS and ET-1/ERK pathways in 5-fluorouracil-induced cardiotoxicity: Modulation by simvastatin

Radwa Nasser Muhammad[1], Nada Mohammed Sallam[1], Hanan Salah El-Abhar[1,2]

[1]Department of Pharmacology and Toxicology, Faculty of Pharmacy, Cairo University, Cairo 11562, Egypt.

[2]Department of Pharmacology & Toxicology, Faculty of Pharmaceutical Sciences and Pharmaceutical Industries, Future University in Egypt, Cairo 11835, Egypt.

Short title: Simvastatin in 5-fluorouracil cardiotoxicity.

Word count: 8100 Manuscript Category: Original Research

Author for Correspondence:
Radwa Nasser Muhammad
Department of Pharmacology and Toxicology
Faculty of pharmacy
Cairo University
Cairo, 11562, Egypt
Email: radwa.nasser@pharma.cu.edu.eg ; Phone: +201118311912

Supplementary figure (S1)

(A) β-Actin

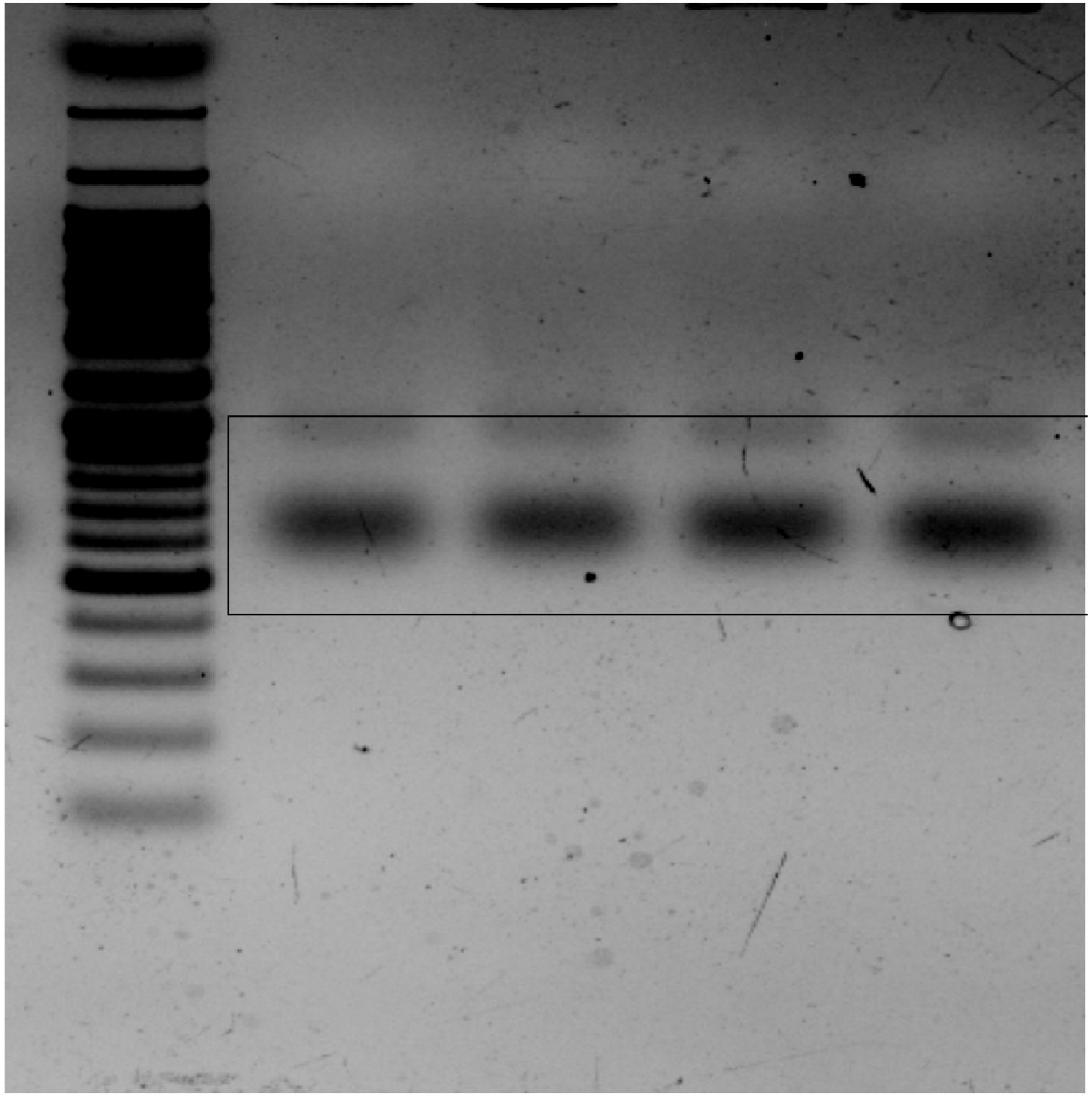

(B) ROCK

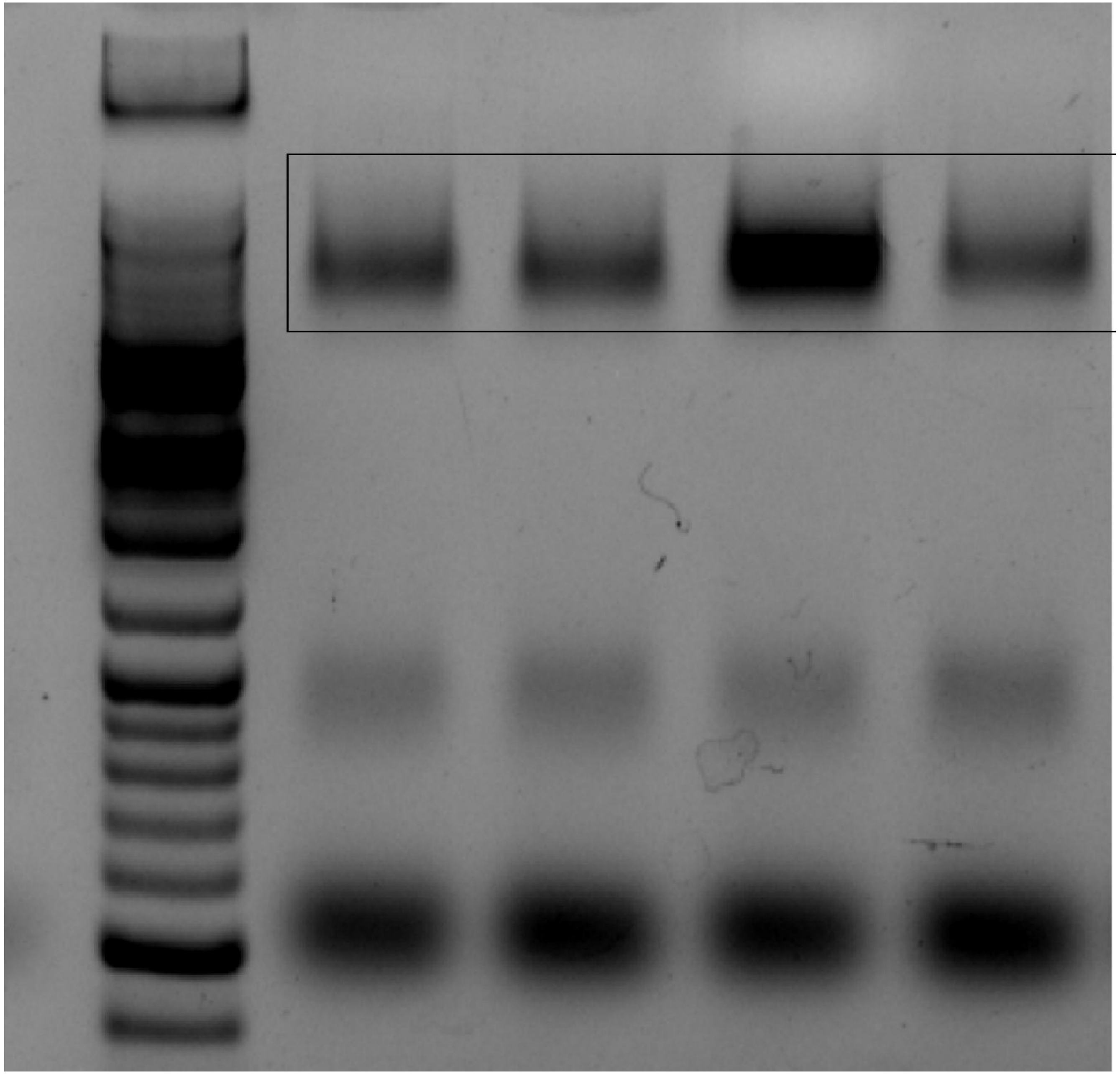

(C) Caspase-3

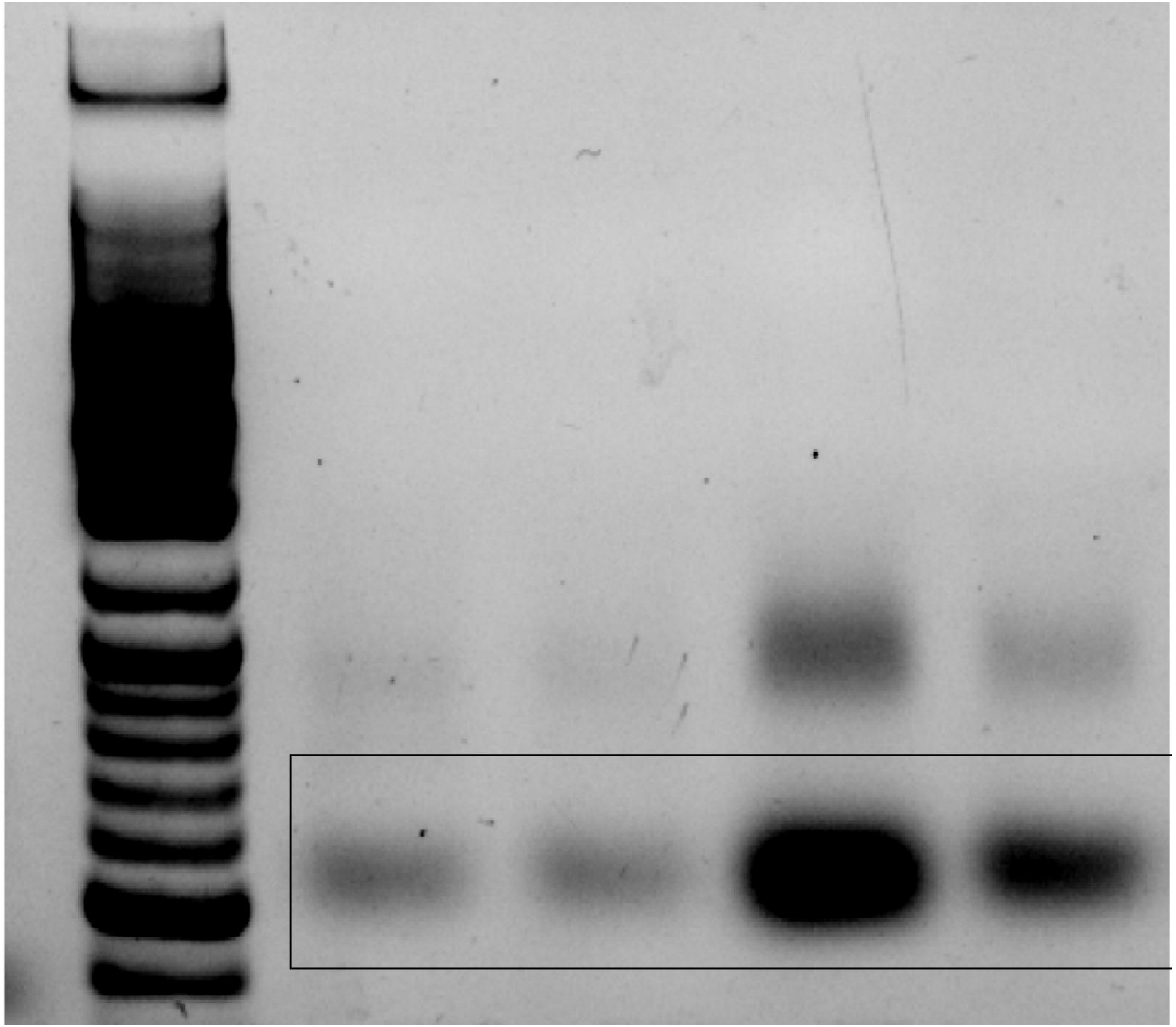

Supplementary figure (S2)

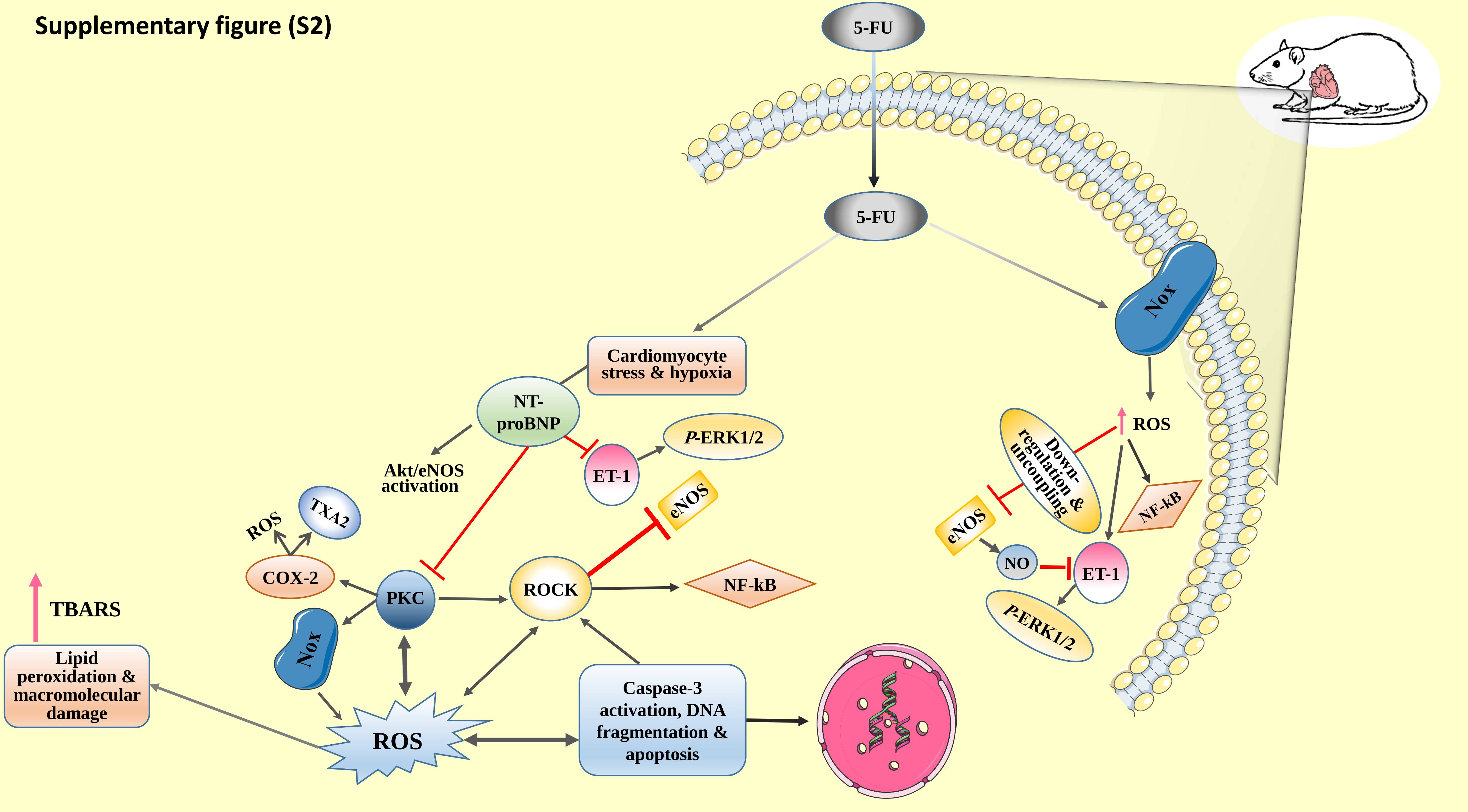

Supplement: Supplementary file 1 — Supplementary Information. [file 41598_2020_71531_MOESM1_ESM.pdf]
