# Supplementary material for: Activated ROCK/Akt/eNOS and ET-1/ERK pathways in 5-fluorouracil-induced cardiotoxicity: modulation by simvastatin
Source: Sci Rep. 2020 Sep 7;10:14693. doi: 10.1038/s41598-020-71531-8 (PMC7477553; doi:10.1038/s41598-020-71531-8)
Supplement: Supplementary file 2 — Supplementary Legend. [file 41598_2020_71531_MOESM2_ESM.docx]

**Supplementary Figure S2:** **A proposed mechanism for 5-FU-induced cardiomyocyte and endothelial cell injury.**

**Abbreviations:** 5-FU, 5-fluorouracil; Akt/PKB, protein kinase B; COX-2, cyclooxygenase-2; eNOS, endothelial nitric oxide synthase; ET-1, endothelin-1; NF-κB, nuclear factor kappa B; NO, nitric oxide; Nox, NADPH oxidase; NT-proBNP, N-terminal B-type natriuretic peptide; *p*-ERK1/2, phosphorylated extracellular signal-regulated kinase 1/2 at Thr202/Tyr204; PKC, protein kinase C; ROCK, rho-kinase; ROS, reactive oxygen species; TBARS, thiobarbituric acid reactive substances; TXA2, thromboxane A2. Grey arrows, activation/ overproduction; Red arrows, suppression/ inhibition; ↑, increase; ↓, decrease.
